# Comparative study between calcium gluconate with diosmin, cabergoline, and cabergoline with diosmin in prevention of ovarian hyperstimulation syndrome in high-risk women undergoing intracytoplasmic sperm injection (ICSI) procedures

**DOI:** 10.3389/fphar.2025.1655866

**Published:** 2025-10-27

**Authors:** Aya M. Abdallah, Amal K. Hussien, Sumaiah J. Alarfaj, Thanaa A. Elmasry, Marwa Kamal, Hatem Sarhan, Mohammed H. Mosbeh, Eman Mohamed Sadek

**Affiliations:** ^1^ Department of Clinical Pharmacy, Faculty of Pharmacy, Minia University, Minia, Egypt; ^2^ Department of Pharmaceutics, Faculty of Pharmacy, Minia University, Minia, Egypt; ^3^ Department of Pharmacy Practice, College of Pharmacy, Princess Nourah Bint Abdulrahman University, Riyadh, Saudi Arabia; ^4^ Pharmacology and Toxicology Department, Faculty of Pharmacy, Tanta University, Tanta, Egypt; ^5^ Pharmacy Practice Department, Faculty of Pharmacy, Sinai University, Arish Branch, Arish, Egypt; ^6^ Department of Clinical Pharmacy, Faculty of Pharmacy, Fayoum University *,* Fayoum *,* Egypt; ^7^ Department of Obstetrics and Gynecology, Faculty of Medicine, Minia University, Minia, Egypt

**Keywords:** ovarian hyperstimulation, cabergoline, calcium gluconate, diosmin, hCG

## Abstract

**Background:**

Ovarian Hyper-stimulation Syndrome (OHSS) is the main iatrogenic problem linked to ovarian stimulation with reproductive medications. Increased vascular permeability is considered the cause of OHSS. It leads to excess fluid flowing from blood vessels into surrounding tissue. Serious consequences include imbalances in electrolytes and impairment of the heart, liver, and kidneys.

**Objectives:**

This study aims to the comparison of the efficacy of calcium gluconate and diosmin combination, cabergoline alone, and a combination of cabergoline and diosmin in preventing OHSS in high-risk individuals undergoing intracytoplasmic sperm injection (ICSI) procedures.

**Methods:**

A prospective, randomized, single-blind, interventional study was carried out on 180 high-risk females for OHSS. They were divided into three groups, each with 60 women: Group A received IV infusion of calcium gluconate and diosmin. Group B received cabergoline alone. Group C received cabergoline and diosmin. Patients were regularly evaluated using clinical examinations and ultrasound to check for signs of OHSS. The main outcome was the frequency of moderate to severe OHSS. Secondary outcomes were the rates of clinical pregnancy, miscarriage, and live birth.

**Results:**

The research demonstrated that there was no significant difference in the prevalence of mild OHSS among the three groups (p-value = 0.91for each). Nevertheless, the three groups showed a notable difference in the occurrence of moderate OHSS (p = 0.044) respectively. Notably, severe ovarian hyper-stimulation syndrome wasn’t observed in Group C. However, 13.3% of patients in Group B and 6.6% in Group A were affected with a highly significant difference (p = 0.007). The chemical and clinical pregnancy rates across the three groups did not demonstrate any statistically significant differences (p-value of 0.53), (p-value of 0.17) correspondingly).

**Conclusion:**

Our study found that using cabergoline and diosmin together in high-risk females undergoing ICSI procedures was significantly more successful in preventing OHSS compared to using calcium gluconate and diosmin or cabergoline alone. This combination did not impact miscarriage, pregnancy rate, or the likelihood of multiple pregnancy.

**Clinical Trial Registration:**

identifier NCT06333691.

## 1 Introduction

Ovarian hyperstimulation syndrome (OHSS) can occur during assisted reproduction as a potential complication of ovarian stimulation. Enlarged ovaries and the sudden movement of fluids from blood vessels to the abdominal cavity characterize it. This can result in bloating, increase the risk of blood clot formation, and reduce blood supply to organs (Tang et al., 2021). Moreover, severe complications such as imbalances in electrolytes and impairment of the heart, liver, and kidneys were observed (Hortu et al., 2021).

The incidence of OHSS was examined in two categories: dopamine agonists and calcium infusion. The meta-analysis found no discernible difference between the two groups (Tang et al., 2021). The infusion of calcium is utilized to reduce the occurrence of OHSS. Increased calcium levels in the blood impede renin release, triggered by cyclic adenosine monophosphate (cAMP), which leads to a decrease in the production of angiotensin-converting enzyme II & reduces vascular endothelial growth factor (VEGF) expression (Lotfalizadeh et al., 2024).

Dopaminergic agonists attach to dopaminergic receptors, which leads to the promotion of endocytosis of the VEGF receptor. This subsequently results in a reduction of neovascularization and vascular permeability (Palomba et al., 2023). Recent research showed that dopaminergic agonists are successful in preventing moderate to severe ovarian hyperstimulation syndrome when compared to no placebo & therapy (Tang et al., 2021).

Recently, diosmin has received a lot of focus due to its potency as a strong venotonic agent. It is a bioflavonoid that can be produced from hesperidin or extracted from various plants. It works by lowering vascular permeability by inhibiting the production of inflammatory agents such as thromboxane and prostaglandin E2 (Saad and Khalil, 2021). The study discovered that utilizing both cabergoline & diosmin in high-risk females with assisted reproductive technology (ART) was effective in preventing ovarian hyperstimulation syndrome compared to utilizing cabergoline alone (Saad and Khalil, 2021).

The current research aims to assess the efficacy of calcium gluconate–diosmin combination, cabergoline alone, and cabergoline-diosmin combination for the prevention of ovarian hyperstimulation in high-risk patients who underwent ICSI procedures.

## 2 Methods

This prospective single blind (outcome assessor) interventional comparative study was carried out in the Nile Centre in Minia Government from October 2022 to February 2024. The study was conducted per the standards of the Declaration of Helsinki and approved by the ethics committee of Minia University’s Faculty of Pharmacy [Approval Number: MPEC (230110)]. The trial was registered on ClinicalTrials.gov with the identifier NCT0633369. Every participant received details about the study, and written informed consent was obtained from each individual.

### 2.1 Inclusion criteria

The participants in the study were women with infertility undergoing intracytoplasmic sperm injection (ICSI) therapy. These cases have been classified as being at high risk for developing ovarian hyperstimulation syndrome. A high-risk case for ovarian hyperstimulation syndrome is determined as a case who has previously had ovarian hyperstimulation syndrome or has more than twenty-four antral follicles in the ovaries throughout a baseline ultrasound (indicating polycystic ovary) or throughout ovarian induction, increased quantity of small follicles (8–12 mm), a high level of Anti-Müllerian Hormone (AMH), a rapidly rising level of serum Estradiol (E2), a high level of serum E2 at hCG trigger (>3,000 pg/mL), or presence of over twenty follicles observed during ultrasound examination on the day of retrieval or more than twenty oocytes retrieved.

### 2.2 Exclusion criteria

The exclusion criteria were patients with hypersensitivity to these agents, patients with endocrine illnesses, e.g., Cushing’s disease, Diabetes Mellitus, asthmatic patients, congenital adrenal hyperplasia, patients with collagen vascular disease, hypercholesterolemic patients, and sickle cell anemia patients, and patients with cardiovascular diseases.

**FIGURE 1:**
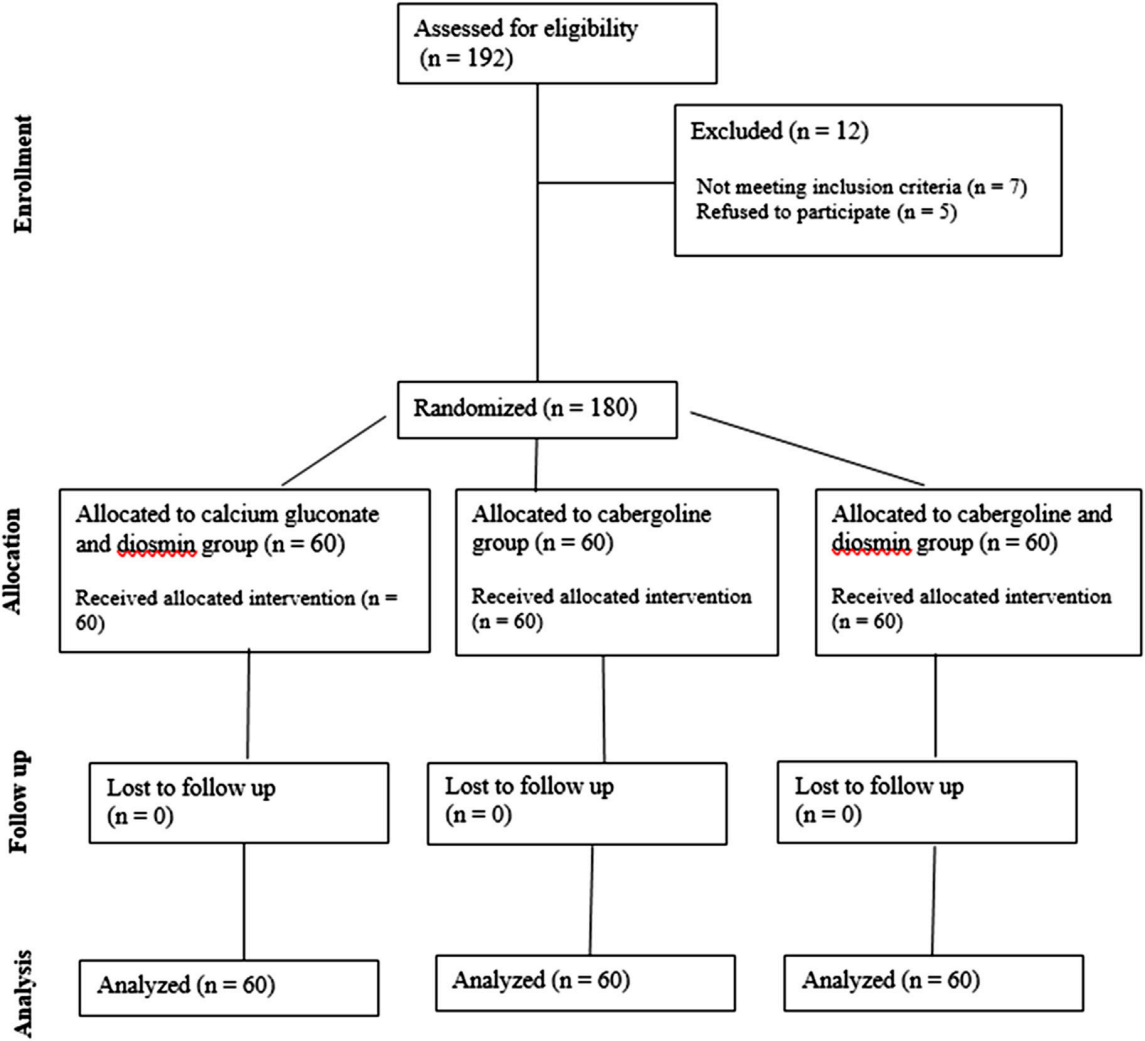
The flow of the patients through the study.

192 patient were evaluated for the eligibility to study (7 patients not meeting inclusion criteria, 5 patients refused to participate). One hundred eighty cases have been recruited for research, and the flow of the patients through the study is outlined in the CONSORT diagram (Figure 1).

### 2.3 Study design

#### 2.3.1 Protocol of stimulation

All cases have been stimulated by a long stimulation protocol utilizing gonadotropin-releasing hormone agonist (GnRHa) (Saad and Khalil, 2021). All patients received:

1. A daily subcutaneous injection of 0.1 mL leuprorelin (Abbott, France) on day 20 of the preceding cycle (to suppress the pituitary),
2. Administering a highly purified form of follicle-stimulating hormone called (Fostimon, produced by IBSA in Switzerland), through an injection into the muscle on the second day of the menstrual cycle. The initial dose ranges from 150–300 μg and is modified daily based on the size of the follicles.
3. A dose of highly pure hCG ten thousand international units (Choriomon, IBSA, Switzerland) was administered when more than two dominant follicles with a diameter of at least 18 mm were detected.

The oocyte was obtained using ultrasound-guided follicular puncture 36 h later. The serum estradiol level of all cases was assessed on the day of hCG administration, and patients with E2 levels 2,500–5,000 pg/mL and follicle size 16 mm or more were selected.

#### 2.3.2 Protocol for OHSS prevention

• Group A: Women received IV infusion of calcium gluconate (Calcionate 10 mL of ten percent calcium gluconate, Memphis) in 200 mL saline within 30 min of ovum pickup and continued for the next 3 days in addition to diosmin two tablets (five hundred milligrams) three times a day for 2 weeks.
• Group B: Women received cabergoline (Dostinex 0.5 mg, Pfizer, Canada, Montreal) orally daily for 8 days after hCG triggering.
• Group C: Female cases were administered diosmin, two tablets (Daflon 500 mg, Servier, France), three times a day for a duration of 2 weeks. Additionally, they were given cabergoline, one tablet of 0.5 mg per day orally for a period of 8 days, starting on the same day as the hCG injection.

### 2.4 Diagnosis of OHSS

OHSS has been confirmed & graded according to the Royal College of Obstetricians & Gynecologists (RCOG, 2016) (Royal College of Obstetricians and Gynaecologists RCOG, 2016) they range from mild to critical women.

• Mild OHSS was characterized by enlargement of ovaries on both sides, slight diarrhea, mild vomiting, or minor nausea.
• Moderate OHSS present with complaints of nausea, vomiting, enlarged ovaries larger than 5 cm, and the presence of ascites.
• Severe OHSS was characterized by tense ascites detected clinically or hydrothorax.
• Critical ovarian hyperstimulation syndrome has been identified based on the presence of tense ascites, significant hydrothorax, hematocrit levels exceeding 0.55, white blood cell count exceeding 25000/mL, anuria or oliguria, thromboembolism, & acute respiratory distress syndrome.

Severe or critical OHSS patients were hospitalized, while mild & moderate OHSS patients were managed in the outpatient setting.

The primary outcome was how often moderate to severe OHSS occurred, whereas the secondary outcome involved the rate of clinical pregnancy, rate of miscarriage, & rate of live birth.

### 2.5 Sample size calculation

The sample size was initially estimated based on prevalence data reported by Li et al. (Li et al., 2020), in which the intervention and control groups demonstrated prevalence rates of 6.8% and 19.4%, respectively. To ensure robustness, this estimation was subsequently confirmed using a priori power analysis with G*Power software (version 3.1.9.7). The calculation assumed a medium effect size (d = 0.4), a significance level of 0.05, statistical power of 0.80, and an allocation ratio of 1:1. The analysis indicated a total required sample size of 200 participants, which was adopted for the present study.

### 2.6 Statistical analysis

The data analysis software utilized was SPSS version 21. Normality was checked using the Shapiro–Wilk test. Depending on distribution, ANOVA or Mann–Whitney tests were applied, and Chi-square was used for categorical data. Significant ANOVA results were further examined using Tukey’s post-hoc test, with p < 0.05 as the threshold for significance.

## 3 Results

The patients’ demographic characteristics illustrated that there were insignificant variations in BMI, age, type, length, and etiology of infertility (Table 1). Moreover, there were insignificant differences among the 3 groups concerning basal FSH, LH, Estrogen level, prolactin, and AMH (Table 2).

**TABLE 1:** Demographic characteristics of women in the three studied groups.

Demographic characteristics	Group A (calcium gluconate + diosmin) N = 60	Group B (cabergoline) N = 60	Group C (cabergoline + diosmin) N = 60	Test	P value
Age (years) Mean ± SD	29.2 ± 5.75	29.5 ± 4.78	28.9 ± 5.36	F = 0.191	0.826
BMI (Kg/m^2^) Mean ± SD	25.16 ± 2.1	24.73 ± 2.49	24.7 ± 3.08	F = 0.593	0.553
Infertility duration (years) Mean ± SD	6.13 ± 4.16	6.5 ± 4.67	5.2 ± 2.9	F = 1.6992	0.185
Type of infertility					
Primary	36 (60%)	30 (50%)	34 (56.7%)	X^2^ = 1.26	0.53
Secondary	24 (40%)	30 (50%)	26 (43.3%)		
Cause of infertility					
tubal	22 (36.7%)	24 (40%)	22 (36.7%)	X^2^ = 5.318	0.503
Male factor	18 (30%)	18 (30%)	18 (30%)		
unexplained	10 (16.7%)	6 (10%)	14 (23.3%)		
more than one factor	10 (16.7%)	12 (20%)	6 (10%)		

P value >0.05: Not significant, P value <0.05 is statistically significant, p <0.001 is highly significant., SD, standard deviation; F, ANOVA test; X^2^, Chi-square; BMI, body mass index.

**TABLE 2:** Hormonal assay among patients in the studied groups.

Hormonal assay	Group A Mean ± SD	Group B Mean ± SD	Group C Mean ± SD	Test	P value
FSH (mIU/mL)	7.57 ± 1.15	7.36 ± 1.39	7.2 ± 1.47	F = 1.144	0.320
LH (mIU/mL)	7.75 ± 1.78	7.32 ± 2.46	6.99 ± 1.38	F = 2.35	0.098
Prolactin (ng/mL)	16 ± 1.86	15 ± 2.8	15 ± 3.4	F = 2.854	0.075
AMH (ng/mL)	2.38 ± 1.15	2.14 ± 1.6	2.16 ± 1.2	F = 0.5997	0.550
Estrogen (E2) (pg/mL)	45 ± 3.15	46 ± 2.3	46 ± 2.6	F = 2.7307	0.067

It can be observed from Table 3 that there were statistically insignificant variations among the studied groups concerning the total number of gonadotropin ampules, stimulation duration, follicles retrieved, fertilization rate, & good-quality embryos.

**TABLE 3:** Outcome of ovarian stimulation among patients in the studied groups.

Outcome of ovarian stimulation	Group A N = sixty Mean ± SD	Group B N = sixty Mean ± SD	Group C N = sixty Mean ± SD	Test	P value
Total dose of gonadotropin used (IU)	21.2 ± 0.84	20.9 ± 1.36	21.2 ± 1.5	F = 1.1238	0.32
Length of gonadotropin stimulation (days)	11.2 ± 0.8	10.93 ± 0.98	11.03 ± 0.96	F = 1.3299	0.267
Number of retrieved follicles	32.3 ± 6.5	30.3 ± 4.9	30.3 ± 7.15	F = 2.0446	0.132
Fertilization rate	70.1 ± 0.44	70.2 ± 0.28	70.1 ± 0.37	F = 1.4674	0.233
Number of good quality embryos	17.2 ± 3.68	16.7 ± 2.15	16.2 ± 3.59	F = 1.4491	0.237

The fertilization rate was determined by calculating the percentage of micro-injected oocytes that developed into two pronuclei (Jawed et al., 2016), Embryos are typically classified as high quality when they achieve the blastocyst stage within 120 h after fertilization. In addition, they should have a fully developed trophectoderm encasing a large fluid-filled cavity and a densely packed group of inner cell mass cells (Ginsburg et al., 2014).

**FIGURE 2:**
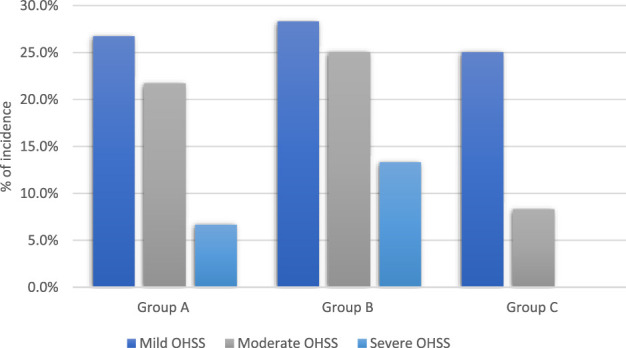
Incidence of OHSS among patients in the studied groups.

The data presented in Table 4 indicate that there were insignificant variances in the occurrence of mild ovarian hyper-stimulation syndrome among the 3 groups (p = 0.91 for each). However, the frequency of moderate ovarian hyper-stimulation syndrome was statistically significant between group A and B, A and C, B and C (p-value of 0.048, 0.04 and 0.01 respectively). Notably, severe ovarian hyper-stimulation syndrome was not observed in Group C. However, it affected 13.3% of patients in group B and 6.6% in group A, with a highly significant difference between group A and B, A and C, B and C (p = 0.047, 0.04 and 0.003 respectivley). Therefore, cabergoline and diosmin combination led to a considerable decrease in the occurrence of severe ovarian hyper-stimulation syndrome, as shown in Figure 2.

**TABLE 4:** Incidence of ovarian hyperstimulation (OHSS) syndrome among patients in the studied groups.

Incidence of ovarian hyperstimulation	Group A (calcium gluconate + diosmin) N = 60	Group B (cabergoline) N = 60	Group C (cabergoline + diosmin) N = 60	P value
Mild OHSS	16 (26.7%)	17 (28.3%)	15 (25%)	0.91
Moderate OHSS	13 (21.7%)	15 (25%)	5 (8.3%)	0.044 P1 = 0.048 P2 = 0.04 P3 = 0.01
Severe OHSS	4 (6.6%)	8 (13.3%)	0 (0%)	0.007 P1 = 0.047 P2 = 0.04 P3 = 0.003

P value >0.05: Not significant, P value <0.05 is statistically significant, p <0.001 is highly significant. P1: Group A vs. Group B, P2: Group A vs. Group C, P3: Group B vs. Group C.

The 2ry outcomes (chemical pregnancy, clinical pregnancy, multiple pregnancy rate, & spontaneous abortion rate) across the three groups didn’t demonstrate any statistically significant distinctions differences as shown in Table 5.

**TABLE 5:** Reproductive outcomes in ICSI cycles among patients in the study groups.

Reproductive outcomes in ICSI cycles	Group A (calcium gluconate + diosmin) N = 60	Group B (cabergoline) N = 60	Group C (cabergoline + diosmin) N = 60	Test	P value
Chemical pregnancy					
Positive	32 (53.3%)	30 (50%)	36 (60%)	X^2^ = 1.254	0.53
Negative	28 (46.7%)	30 (50%)	24 (40%)		
Clinical pregnancy					
Positive	26 (43.3%)	20 (33.3%)	30 (50%)	X^2^ = 3.462	0.17
Negative	34 (56.7%)	40 (66.7%)	30 (50%)		
Multiple pregnancy rate	5/60 (8.3%)	4/60 (6.6%)	4/60 (6.6%)	F = 2.6242	0.075
Spontaneous abortion rate	7/60 (11.6%)	7/60 (11.6%)	6/60 (10%)	F = 2.5221	0.083

P value >0.05: Not significant, P value <0.05 is statistically significant, p <0.001 is highly significant. SD, standard deviation; F, ANOVA test; X2, chi-square.

A chemical pregnancy is an early miscarriage that occurs within the first 5 weeks of gestation, prior to the pregnancy being visible on an ultrasound (Cleveland, 2025). A clinical pregnancy refers to a pregnancy that has continued for 6 weeks (or 42 days) following the start of the last menstrual period, and a hCG test verifies it (Wang et al., 2003).

## 4 Discussion

To our knowledge, this is the first clinical research to assess the efficacy of calcium gluconate with diosmin in the prevention of OHSS in ICSI procedure. The research found that the prevalence of mild OHSS was similar across all three groups, showing a p-value of 0.91 for each. However, there was a significant difference in the rates of moderate OHSS, with a p-value of 0.044. Additionally, severe OHSS was not present in Group C. In contrast, 13.3% of patients in Group B and 6.6% in Group A experienced severe OHSS with a highly significant difference, indicated by a p-value of 0.007.

Diosmin is an affordable, well-tolerated drug (Saad and Mohamed, 2017). During pregnancy, diosmin has a satisfactory safety profile. There have been no reports of embryotoxicity or other notable effects on reproductive function from using Diosmin during pregnancy. Additionally, the transfer of Diosmin through the placenta and into breast milk is minimal (Meyer, 1994). Research was undertaken involving 202 individuals who were susceptible to developing ovarian hyperstimulation during an *in vitro* fertilization (IVF) procedure. The cases were separated at random into two groups. Group I, consisting of ninety-eight cases, was given an intravenous calcium gluconate infusion, while Group II, consisting of 104 cases, was given cabergoline. The incidence of ovarian hyperstimulation syndrome was observed in only nine cases (9.2 percent) in the calcium infusion group, whereas sixteen cases (15.4 percent) of patients who received cabergoline had the condition. Nevertheless, the difference in the incidence of ovarian hyperstimulation syndrome between the two groups was found to be statistically insignificant. Out of nine cases in the calcium infusion group who had the problem, only one had severe ovarian hyperstimulation syndrome, two cases had moderate ovarian hyperstimulation syndrome, and six cases had mild OHSS. In contrast, the cabergoline group had two females diagnosed with severe ovarian hyperstimulation syndrome, four cases with moderate ovarian hyperstimulation, and ten cases with mild OHSS. Each of the three women, according to both research groups, had severe ovarian hyperstimulation syndrome and had to be admitted to the hospital (Naredi and Karunakaran, 2013).

In addition, researchers performed an investigation including 170 cases, which were separated into two groups: the cabergoline group & the calcium gluconate group. The study revealed that six cases in the cabergoline group & eight cases in the calcium gluconate group had moderate ovarian hyperstimulation syndrome. Severe OHSS occurred in one patient from each group. There was an insignificant difference in moderate/severe ovarian hyperstimulation occurrence among the two groups (P value = 0.599) (Fouda et al., 2022).

A study conducted on 200 women who were at high risk for developing OHSS were randomly allocated into two groups. Group A (Diosmin group, 100 women) and group B (Cabergoline group, 100 women) found that there was a statistically significant reduction (P = 0.005) in the incidence of OHSS in the diosmin group (12%) compared to cabergoline group (28%). The number of severe OHSS cases in the cabergoline group (n = 13) was significantly higher (P = 0.003) than in the diosmin group (n = 2) (Saad and Mohamed, 2017).

Calcium gluconate reduces renin secretion and subsequently lowers the levels of angiotensin II and VEGF (Lotfalizadeh et al., 2024). Diosmin has the advantage of prolonging the duration of contractions, even at high temperatures, enhancing venous tension. It specifically targets veins and does not impact the arterial system. Diosmin also decreases the adhesion and movement of leukocytes to vascular endothelial cells and restrains the release of inflammatory substances, thereby lowering capillary permeability (Li et al., 2020). Gurgan et al. investigated the effect of Calcium injections on the rates of ovarian hyperstimulation syndrome (OHSS) in women with polycystic ovary syndrome. The findings indicated that administering intravenous Calcium notably decreased both the occurrence and severity of OHSS, with just 3.6% of treated patients suffering from mild cases, in contrast to 16.2% in the control group. Additionally, no significant side effects were observed (Gurgan et al., 2011).

In addition, another study aimed to assess the efficacy of combining diosmin and cabergoline compared to using cabergoline alone in preventing OHSS in high-risk cases completing assisted reproductive method cycles. One hundred women who were at a high risk of having OHSS participated in the trial. These females were divided into two groups: Group A, consisting of fifty women who received a combination of diosmin and cabergoline, & Group B, consisting of fifty women who received only cabergoline. The study findings showed a statistically significant decrease in ovarian hyperstimulation occurrence (P = 0.005) in the group receiving both diosmin & cabergoline compared to the group receiving only cabergoline (group B). Their findings indicated that the inclusion of diosmin along with cabergoline was more in its ability to avoid severe ovarian hyperstimulation syndrome & reduce the occurrence rates of ovarian hyperstimulation syndrome compared to cabergoline alone, particularly in high-risk cases (Saad and Khalil, 2021). Our study introduced a third group receiving calcium gluconate with diosmin to compare its efficacy with that of cabergoline and diosmin combination. We found that the combination of Cabergoline and diosmin is more effective in preventing OHSS in high-risk patients, followed by the combination of calcium gluconate and diosmin, and then Cabergoline alone.

Considering reproductive outcomes, research revealed that the implantation, clinical pregnancy, & ongoing pregnancy rates were similar in both groups (P = 0.736), which supports our results (p > 0.05 for each) (Fouda et al., 2022).

Also, another study reported that there was no significant difference in clinical pregnancy rate among both groups (diosmin+cabergoline) group and cabergoline group (P = 0.87) (Saad and Khalil, 2021).

Future research should directly compare the efficacy of calcium gluconate and cabergoline alone and with diosmin in a larger sample size to assess the efficacy of each group compared to others.

## 5 Conclusion

From the results of this investigation, it can be concluded that Cabergoline - Diosmin combination is more effective in preventing OHSS in high-risk patients undergoing ICSI procedures followed by Calcium gluconate - Diosmin combination, then Cabergoline alone without affecting miscarriage, pregnancy rate, or multiple pregnancy. We recommend conducting further studies on a large scale to confirm our results.

## Data Availability

The raw data supporting the conclusions of this article will be made available by the authors, without undue reservation.
